# Atomic-scale study of the amorphous-to-crystalline phase transition mechanism in GeTe thin films

**DOI:** 10.1038/s41598-017-08275-5

**Published:** 2017-08-15

**Authors:** R. Mantovan, R. Fallica, A. Mokhles Gerami, T. E. Mølholt, C. Wiemer, M. Longo, H. P. Gunnlaugsson, K. Johnston, H. Masenda, D. Naidoo, M. Ncube, K. Bharuth-Ram, M. Fanciulli, H. P. Gislason, G. Langouche, S. Ólafsson, G. Weyer

**Affiliations:** 1 Laboratorio MDM, IMM-CNR, Via Olivetti 2, 20864 Agrate Brianza (MB), Italy; 2 Physics Department, ISOLDE/CERN, Geneva 23, Switzerland; 3 Dept. of Physics, K. N. Toosi University of Technology, P.O. Box 15875-4416 Tehran, Iran; 4 Science Institute, University of Iceland, Dunhaga 3, 107 Reykjavík, Iceland; 5 School of Physics, University of the Witwatersrand, Johannesburg, 2050 South Africa; 6 Durban University of Technology, Durban, 4000 South Africa; 7 School of Chemistry and Physics, University of KwaZulu-Natal, Durban, 4000 South Africa; 8 Dipartimento di Scienza dei Materiali, Università di Milano Bicocca, Milano, Italy; 9 KU Leuven, Instituut voor Kern-en Stralings Fysika, B-3001 Leuven, Belgium; 10 Department of Physics and Astronomy, Aarhus University, Aarhus C, Denmark; 11 Laboratory for Micro- and Nanotechnology, Paul Scherrer Institute, 5232 Villigen PSI, Switzerland

## Abstract

The underlying mechanism driving the structural amorphous-to-crystalline transition in Group VI chalcogenides is still a matter of debate even in the simplest GeTe system. We exploit the extreme sensitivity of ^57^Fe emission Mössbauer spectroscopy, following dilute implantation of ^57^Mn (T½ = 1.5 min) at ISOLDE/CERN, to study the electronic charge distribution in the immediate vicinity of the ^57^Fe probe substituting Ge (Fe_Ge_), and to interrogate the local environment of Fe_Ge_ over the amorphous-crystalline phase transition in GeTe thin films. Our results show that the local structure of as-sputtered amorphous GeTe is a combination of tetrahedral and defect-octahedral sites. The main effect of the crystallization is the conversion from tetrahedral to defect-free octahedral sites. We discover that only the tetrahedral fraction in amorphous GeTe participates to the change of the Fe_Ge_-Te chemical bonds, with a net electronic charge density transfer of  ~ 1.6 e/a_0_ between Fe_Ge_ and neighboring Te atoms. This charge transfer accounts for a lowering of the covalent character during crystallization. The results are corroborated by theoretical calculations within the framework of density functional theory. The observed atomic-scale chemical-structural changes are directly connected to the macroscopic phase transition and resistivity switch of GeTe thin films.

## Introduction

Chalcogenide materials are characterized by fast and reversible phase transitions, which are typically accompanied by orders of magnitude variations in their electrical resistivity, as well as by large differences in their optical reflectivity, making them extraordinarily important for non-volatile memory applications^1^. Typically, such phase transitions are correlated with electrically/optically-induced fast and reversible switching between amorphous and crystalline phases above room temperature (RT). The interest in GeTe has recently been revived from both fundamental and technological points of view, in different fields ranging from phase-change memories to spintronics^2–15^.

Even though several chalcogenide compounds (for example, Ge_2_Sb_2_Te_5_) are already employed in data storage devices, the microscopic amorphous-to-crystalline transition mechanism in even the simple GeTe system is still an open question^16–21^. In particular, Kolobov *et al*., having recently reported on simulations of the phase change in GeTe, pointed out the current lack of information about the changes in the Ge-Te chemical bond character during the phase transition^21^. Indeed, only few experimental methods are suitable to probe the local electronic structure changes around Ge in GeTe across structural transitions, and results so far are contradictory. Based on extended X-ray absorption fine structure (EXAFS) measurements, some groups showed that, upon amorphization, the average coordination of Ge atoms decreases from six-fold in the crystalline phase (c-GeTe) to four-fold in the amorphous state (a-GeTe)^17, 18^. Other groups, though, also on the basis of EXAFS results, proposed alternative scenarios^22^. Based on X-ray photoelectron spectroscopy (XPS), Betts *et al*.^23^ observed a relatively large shift in the Ge 3*d* level upon crystallization, which was attributed to a covalent-to-ionic change of the Ge-Te chemical bonding without a strong change in the bond lengths; on the other hand, Shevhik *et al*. concluded the opposite, i.e. that the phase change in GeTe has to be attributed mainly to local symmetry changes with no change in the charge density around Ge^24^. The latter interpretation has been supported by synchrotron-based XPS experiments^25^, while different groups have reported changes in the electronic structure of a-GeTe and c-GeTe^26^. The evident controversy in the interpretation of XPS results underlines the need for an experimental method more sensitive to the very small valence state changes occurring at the Ge site during the amorphous-to-crystalline GeTe phase transition. In particular, while the structure of c-GeTe seems quite well understood, the main questions that are left concern the local structure of a-GeTe and, particularly, the mechanisms driving the a-GeTe to c-GeTe phase transition at the atomic-scale^21^. Andrikopoulos *et al*. have applied Raman scattering to show that the structure of a-GeTe contains only tetrahedral GeTe_4−n_Ge_n_, species (n = 0, 1, 2, 3, 4), whereas Te-Te bonds are absent^27^. They have observed that the n = 0 case gradually dominates when increasing the annealing temperature (before the phase transition), finally driving the phase change to c-GeTe.

Mössbauer spectroscopy (MS) is an ideal tool for measuring local variations of charge density and symmetry around the Mössbauer-active probe in materials experiencing macroscopic phase transformations, and ^119^Sn and ^125^Te MS have been previously conducted on both glassy and c-GeTe compounds^28–33^. By ^119^Sn MS at Ge sites, the local structure of amorphous Ge_x_Te_1−x_ (x ≤ 0.2) alloys has been described with the co-existence of tetrahedral and the so-called defect-octahedral (i.e. Ge in an octahedral configuration with two nearest neighbors, nn, Te vacancies) local configurations^33^. Again by ^119^Sn MS, it has been shown that Ge atoms in a-GeTe are tetrahedrally coordinated with the Te nn in a covalent-type of bonding; while, upon crystallization, Ge acquires the 2+ charge state, as expected in the c-GeTe crystal, with Ge surrounded by six Te atoms as nn^28–30^. The isomer shift at ^125^Te sites in amorphous and crystalline GeTe has been reported to be the same within the experimental error, while a strong change in the electric field gradient has been observed^31^.

Here, we present results obtained by temperature-dependent ^57^Fe emission Mössbauer spectroscopy (eMS) in GeTe, as performed at the radioactive ion beam facility ISOLDE at CERN. Such an experimental method is sensitive to the nuclear hyperfine interactions between the ^57^Fe nuclei and their nn and next nn ions (nnn). In particular, eMS is used to investigate the Fe site location in GeTe following the implantation of ^57^Mn, and to determine the atomic scale mechanisms at the basis of the phase change occurring in GeTe upon thermal annealing. When compared to ^119^Sn and ^125^Te MS experiments^28–33^, ^57^Fe MS is characterized by a higher sensitivity to potential small variations in the local valence states of the probe ions and the local symmetry around the Mössbauer probe due to the smaller intrinsic linewidth of the 14.4 keV transition^34^. A special feature of the eMS approach is that the implantation fluence is kept very low (10^10-12^ ions/cm^2^), corresponding to a concentration of 10^−4^–10^−3^ at.%. This assures single ion implantation, without overlapping damage cascades, and rules out any prospect of Mn/Fe precipitation. The eMS measurements are done at the implantation temperature, and the atomic-scale information is obtained with Mn/Fe probes at rest, 1.5 min. after the implantation. More importantly, eMS allows *in situ* monitoring of the local changes occurring *during* and *across* the a-GeTe to c-GeTe phase transition. The experimental approach in this work is unique, since eMS was carried out on thin films of GeTe which have been previously characterized by temperature-dependent resistivity measurement, whose preparation is described in the methods sections (see also ref. 35 and references therein). By doing so, we seek a correlation between the resistivity switching and the thermally induced crystallization tracked at the atomic scale in the *in-situ* eMS study.

Our experimental findings are corroborated by simulations based on first principles calculations in the framework of density functional theory (DFT).

## Results and Discussion

### Basic properties of the GeTe films

**Figure 1:**
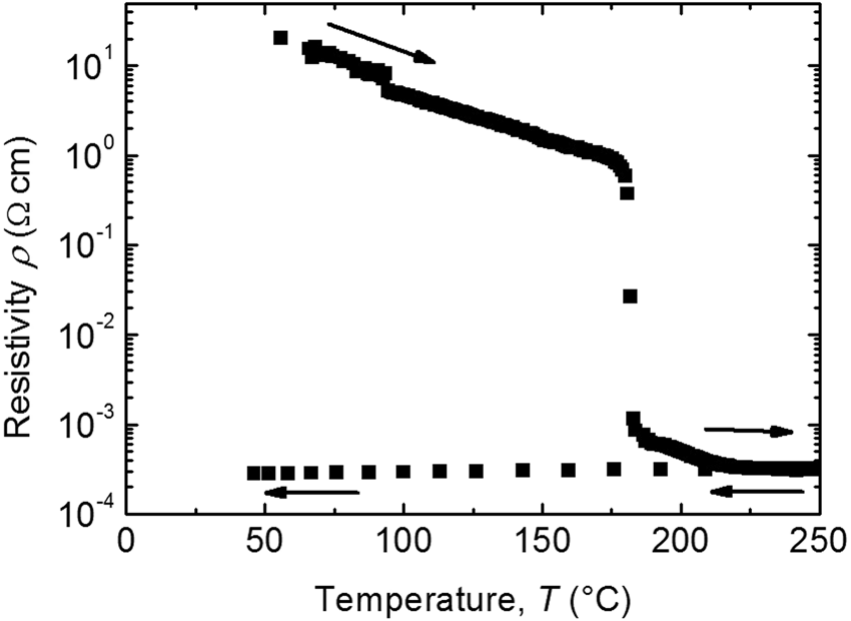
Electrical resistivity changes in GeTe-1 following thermal annealing, where the amorphous-to- crystalline phase transition is evident at 180 °C.

Two samples, labelled GeTe-1 and GeTe-2, were cut from the same wafer and are the subject of the present study. The electrical resistivity (*ρ*) of sample GeTe-1 was measured as a function of temperature in a vacuum chamber, while sample GeTe-2 was used for *in situ* temperature-dependent eMS measurements. Figure 1 shows the resistivity of the GeTe-1 sample, as recorded during the thermal annealing. The sample is initially in its amorphous state, showing a resistivity *ρ* ≈ 10 Ωcm. Upon heating, the resistivity sharply drops at the transition temperature T_ac_ ≈ 180 °C as a result of the amorphous-to-crystalline phase transition, and the crystalline structure is retained until the end of the thermal treatment at 250 °C, when complete transformation is achieved. Once crystallized, the film remains in a low resistivity state down to RT, since the re-amorphization requires melting followed by fast quenching. The resistivity values in both the crystalline and amorphous states are in agreement with those previously reported for GeTe thin films^35–37^.

**Figure 2:**
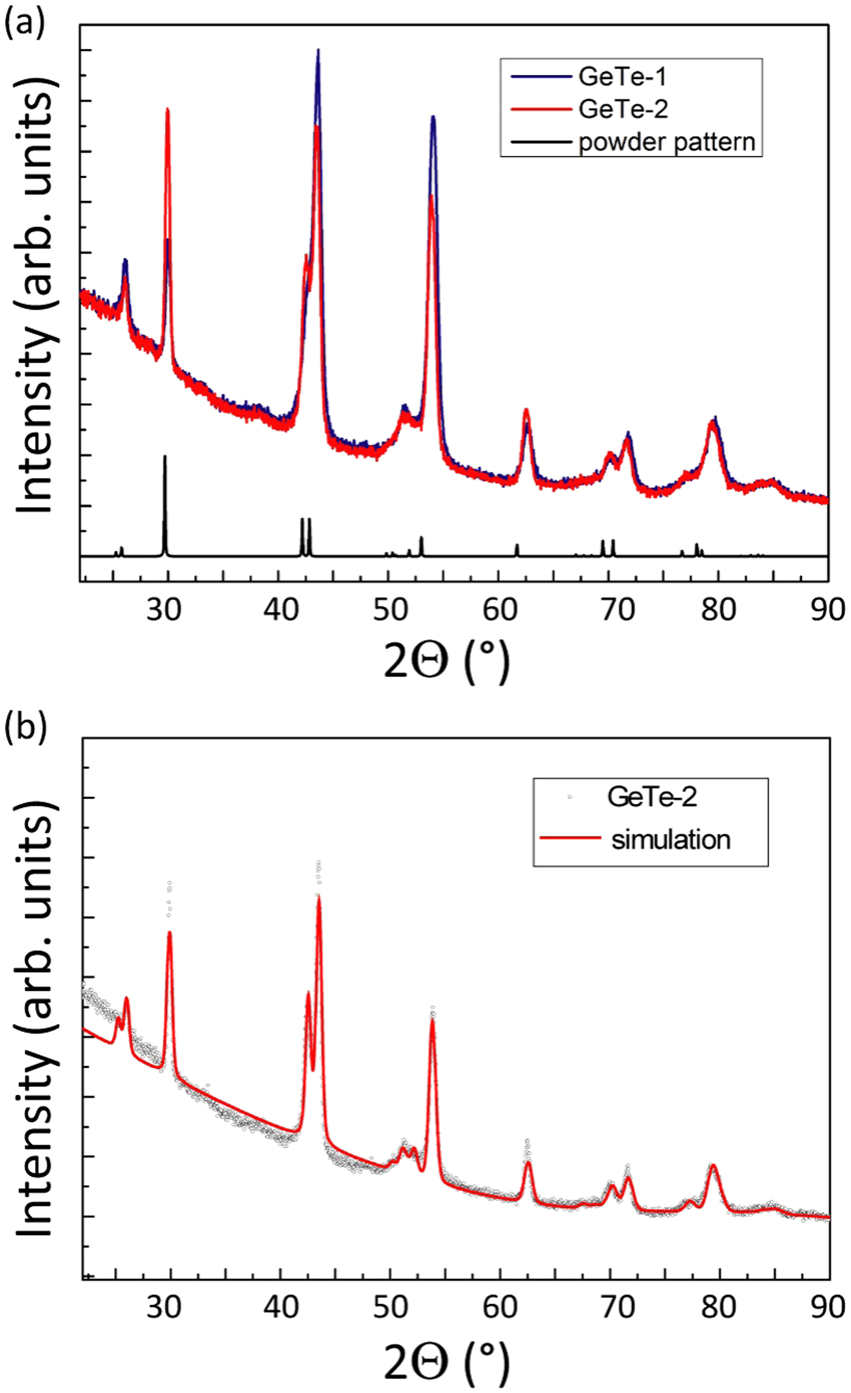
(**a**) GIXRD spectra of GeTe-1 and GeTe-2 samples after thermal annealing above the amorphous-to-crystalline phase transition. (**b**) Fitting of the GeTe-2 crystallized sample with the pattern expected for the rhombohedral R3m:H structure of GeTe.

GIXRD was performed on the two GeTe samples following thermal annealing performed in the Van der Pauw set up on GeTe-1 and during eMS measurements on GeTe-2. Both samples were found to crystallize in the rhombohedral structure R3m:H of GeTe, as shown in Fig. 2(a). This is the expected distorted NaCl structure of GeTe below 670 K^38^. The small variation of the diffracted intensity may evidence a slight variation of the preferential orientation of the crystallites and/or a different structure factor. The lattice parameters were extracted from the Rietveld refinement of the diffraction spectrum of sample GeTe-2, with an arbitrary texture and imposing a micro-strain of 1%. Figure 2(b) shows the obtained simulation within the whole explored 2Θ range. The extracted lattice parameters are *a* = 4.15 Å and *c* = 10.51 Å, which are slightly lower than those reported for stable and stoichiometric GeTe (*a* = 4.21 Å, *c* = 10.60 Å)^39, 40^.

### ^57^Fe eMS in GeTe during *in situ* annealing

**Figure 3:**
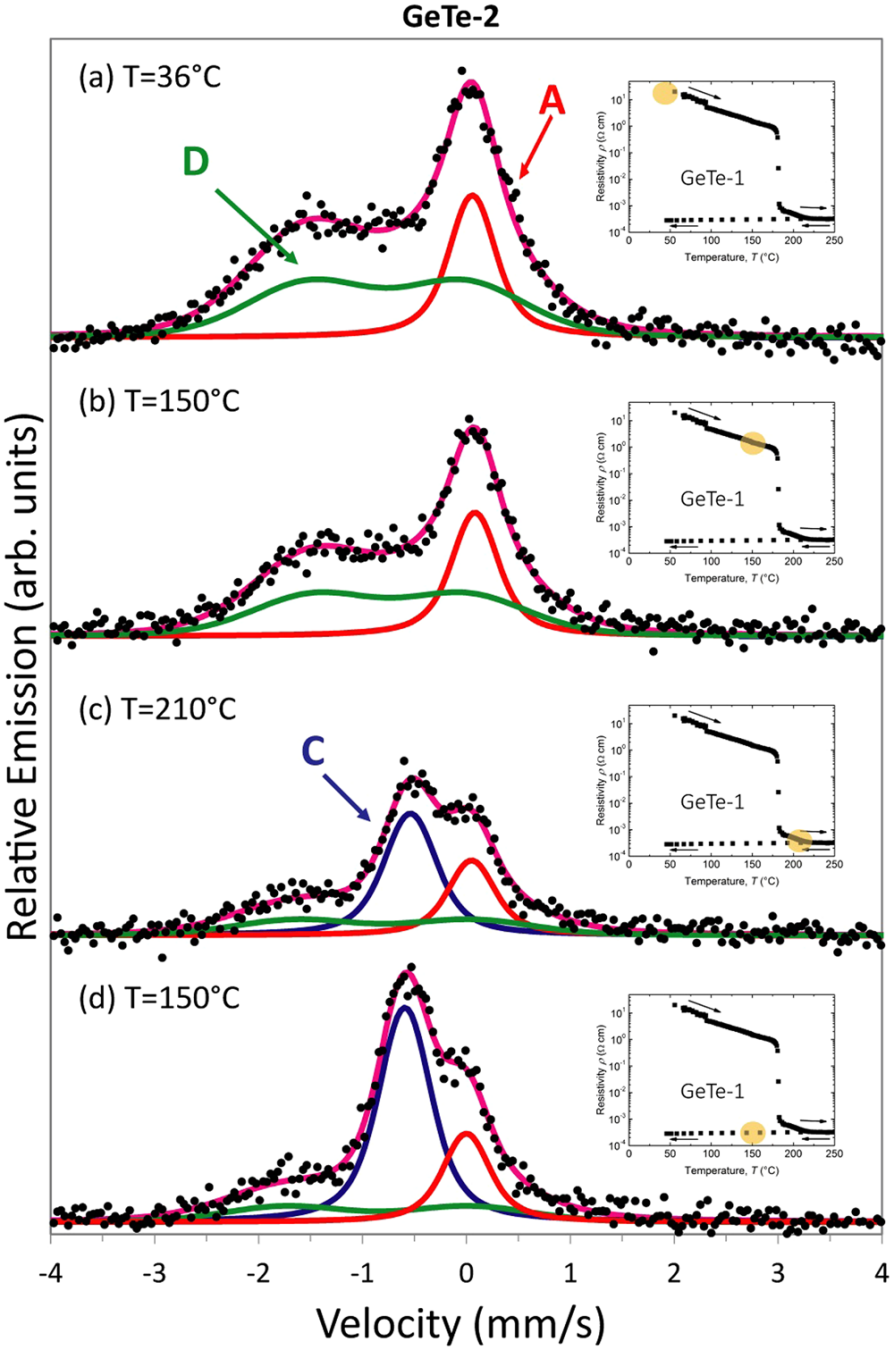
^57^Fe emission Mössbauer spectra obtained on the GeTe-2 held at the temperatures indicated. The purple solid line is the sum of the Lorentzian A and C single lines and the Voigt lineshape quadrupole-doublet D. The insets show the corresponding resistivity state as measured in GeTe-1.

Results of our resistivity measurements (Fig. 1) on sample GeTe-1 show that the amorphous-to-crystalline phase change occurs at T_ac_≈180 °C. Hence, the eMS measurements on the as-grown (amorphous) sample GeTe-2 were conducted at four stages: (a) implantation and measurement at 36 °C; (b) implantation and measurement at 150 °C (i.e. 30 °C below T_ac_); (c) implantation and measurements at 210 °C (i.e 30 °C above T_ac_); (d) implantation and measurements back to 150 °C. The extremely low total concentration of the implanted ions makes the ^57^Fe nuclei a local probe of the macroscopic a-GeTe to c-GeTe structural transition. The respective spectra are presented in Fig. 3(a)–(d). Insets in Fig. 3 report the resistivity curve of the twin GeTe-1 thin film, with the dot markers indicating the corresponding temperatures at which eMS was carried out in GeTe-2.

Before the phase transition in GeTe-2, the eMS spectra are interpreted in terms of two components, labelled A (Lorentzian single line) and D (Voigt line shape quadrupole-doublet), while following the phase transition in both GeTe-1 and GeTe-2, the eMS data are fitted by including the additional single line C. Both A and C components show unresolved quadrupole splitting (Δ*E*_*Q*_ < 0.1 mm/s). The fitting of all the eMS spectra of crystallized GeTe-2 and GeTe-1 was conducted simultaneously, by forcing the isomer shifts of all the components to follow the second order Doppler shift^41^. The quadrupole splitting of the D component showed the typical T^3/2^ temperature dependence as observed for damage components in group IV semiconductors^42^, suggesting a highly disordered local Fe environment, as also manifested with quite large linewidth. Table 1 summarizes the Mössbauer parameters at RT of the identified A, C and D components: isomer shift (*δ)*, quadrupole splitting (Δ*E*_*Q*_) and *σ*_*free*,_ the additional Gaussian broadening of the linewidth (see Methods).

**Table 1:** Mössbauer parameters at RT for the C, A, and D components, as determined by fitting the eMS data of GeTe-2, being: *δ* the isomer shift, *ΔΕ*_*Q*_ the quadrupole splitting, and *σ*_*free*_ is the additional Gaussian broadening free to vary in the fitting procedure.

**Mössbauer parameters (mm/s)**	**C**	**A**	**D**
*δ*	+0.63(1)	−0.057(10)	+0.768(15)
*ΔE*_*Q*_	0	0	1.490(19)
*σ*_*free*_	0.167(6)	0.137(9)	0.52(1)

The eMS spectrum obtained following implantation at 150 °C (Fig. 3(b)) does not show any major changes compared with the 36 °C measurement (Fig. 3(a)), with only the D component showing a slightly lower relative intensity. On the other hand, the spectrum collected at 210 °C (Fig. 3(c)) shows major changes once T_ac_ is passed: the relative intensity of the A component is drastically reduced compared to that observed at 150 °C and the spectrum is dominated, instead, by the new single line C, with a different value of the isomer shift *δ* (Fig. 3c). The change in isomer shift accompanying the transformation of the spectral component A in a-GeTe to the C component in the c-GeTe, corresponds to an energy change of Δ*E* = 3.715 × 10^−8^ eV. The relative intensity of the D component also drops across the phase transition, without displaying any change of the isomer shift. After lowering the temperature to 150 °C (Fig. 3(d)), the eMS spectrum shows that the amorphous structure does not recover, consistently with what shown by the resistivity measurements. However, the full thermal budget furnished to the system, enhances the A to C transformation (see Supplementary Information).

**Figure 4:**
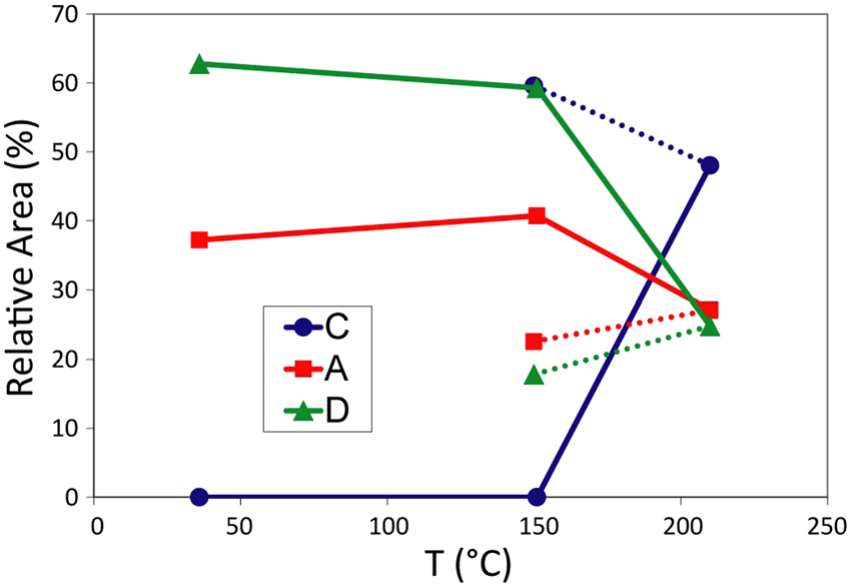
Evolution of spectral areas in the eMS spectra of GeTe-2 as a function of the temperature.

Figure 4 shows the variation with temperature of relative area intensities of the spectral components A, C and D. A 20% fraction of the Fe atoms remains in the A-type of spectral component, even after the implantation and measurement above T_ac_.

The eMS measurements of GeTe-1 were conducted at 36 and 150 °C, following the resistivity measurement depicted in Fig. 1 (see Supplementary Information). They show the dominating C component already at 36 °C, as expected after crystallization occurring during the Van der Pauw measurements (Fig. 1), where the temperature was higher than T_ac_.

### Calculation of Fe hyperfine parameters in GeTe

In order to proceed with the lattice site assignments, and to elucidate the configurational changes occurring across the a–c phase transition, six different configurations were simulated. I, II): Fe substituting Ge (Fe_Ge_) surrounded by six and four Te atoms, as nn in c-GeTe and a-GeTe, respectively; III, IV): Fe substituting Ge surrounded by six Te atoms with an additional one and two Te vacancies in c-GeTe, respectively; V, VI): Fe substituting Te surrounded by six and four Ge atoms, as nn in c-GeTe and a-GeTe, respectively.

**Figure 5:**
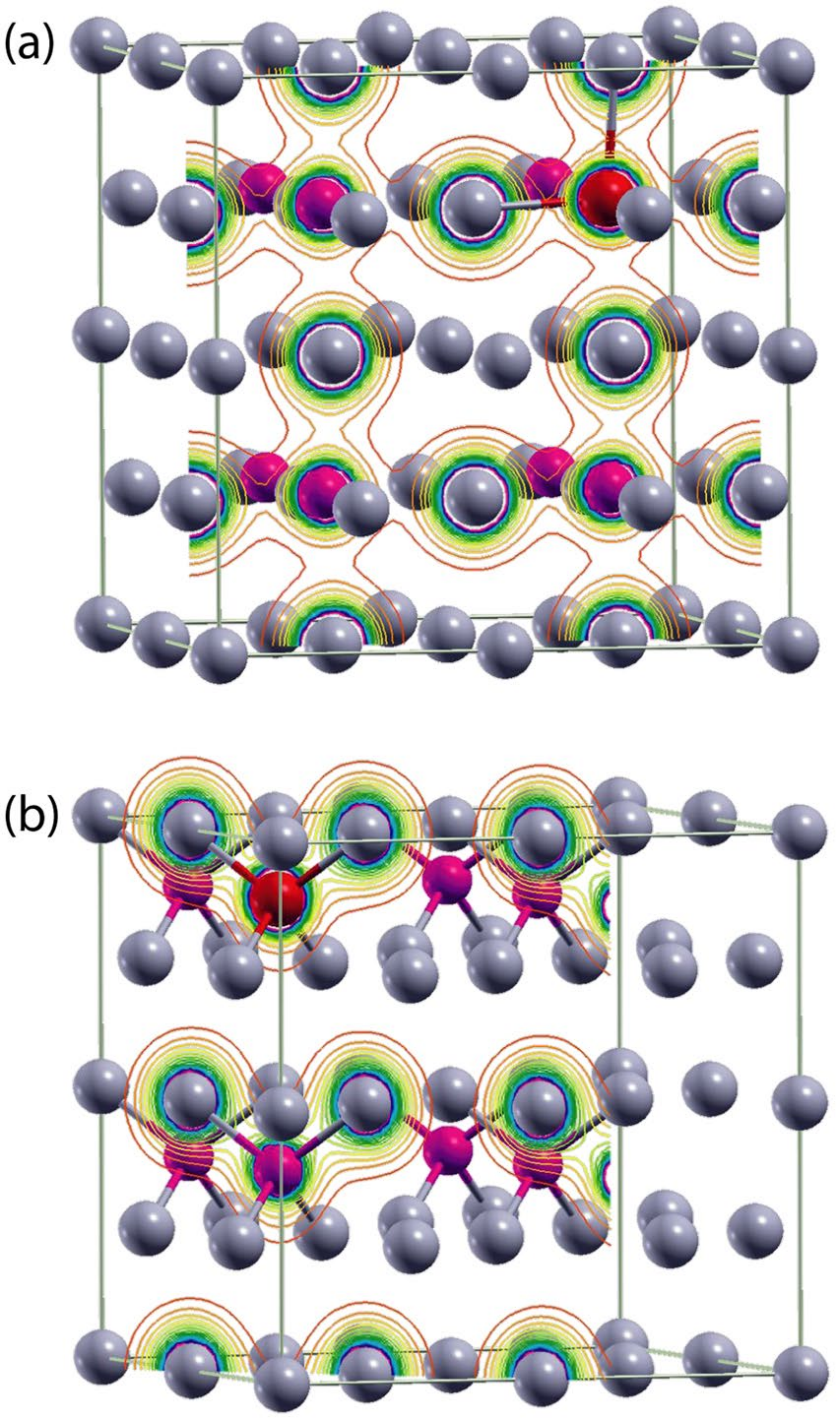
Local structure around Fe_Ge_ in a 2 × 2 × 2 supercell of GeTe in (**a**) the rhombohedral structure of c-GeTe and in (**b**) a-GeTe, where local configuration around Fe_Ge_ is assumed tetrahedral. Red spheres indicate the Fe impurity substituting Ge (purple spheres) in the two configurations, where grey spheres represent the Te atoms.

Figure 5(a) shows configuration I): Fe_Ge_ in an octahedral configuration in the rhombohedral structure (space group R3m) formed by a 2 × 2 × 2 supercell of c-GeTe^17^, with lattice parameters of 6.02 Å^43^, six-fold coordinated by Te with three short (2.83 Å) and three long (3.15 Å) bond-lengths^17, 44^. Figure 5(b) shows configuration II): Fe_Ge_ in a fourfold tetrahedral coordination with the Fe_Ge_-Te distance in the unit cell reduced to 2.5 Å. To simulate the tetrahedral amorphous structure, we forced consistency between the obtained lattice parameters with those calculated from the interatomic distances obtained by EXAFS analysis in a-GeTe^17^.

**Figure 6:**
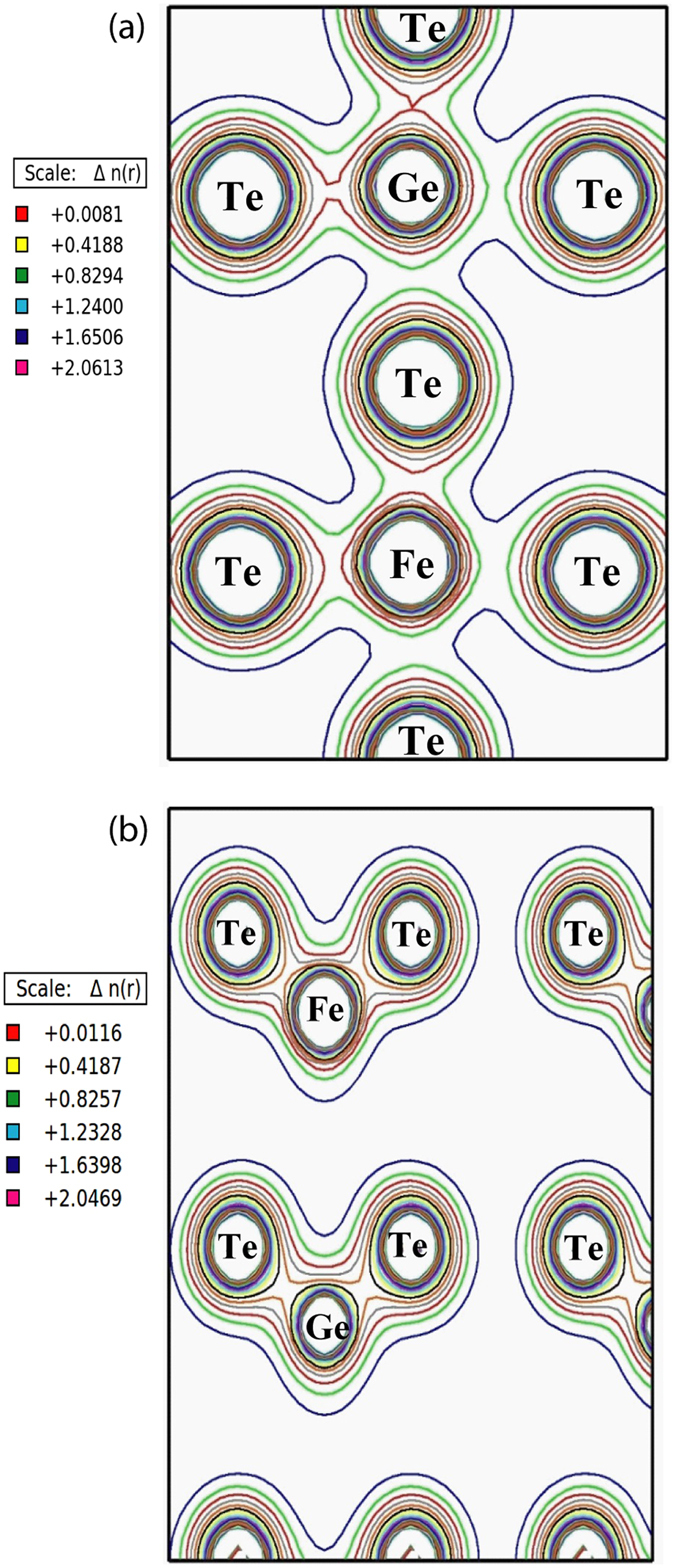
Charge density distribution of Fe-doped GeTe at (**a**) (100) planes of c-GeTe and (**b**) (111) planes of a-GeTe.

Figure 6 shows the charge densities corresponding to the configurations I,II) depicted in Fig. 5, which have been calculated in order to monitor the Fe, Ge and Te valence electron states and charge transfer properties in these c-GeTe and a-GeTe phases. The legends in Fig. 6 indicate the magnitude of the charge density *Δn(r)* (same color code as in Fig. 5). The charge densities around the Fe and Te atoms are mainly formed by *d* and *p* orbital states, respectively. Clearly, there is a higher degree of covalency along the Fe_Ge_-Te bonding in the amorphous case (Fig. 6(b)) than in the crystalline state (Fig. 6(a)).

Table 2 summarizes the *δ*, *V*_*ZZ*_, and *ΔE*_*Q*_ values (see Methods), for all of the six considered configurations.

**Table 2:** DFT calculated electric field gradient (*V*_*zz*_) and Mössbauer parameters δ and *ΔΕ*_*Q*_ for Fe at Ge and Te sites in GeTe, in the indicated symmetry structure. For Fe at the Ge site, the situation in which Te is replaced with 1 and 2 vacancies in c-GeTe is also simulated.

**Local configuration**		**V**_**zz**_ **(x10**_21_ **V/m** ^**2**^ **)**	**δ(mm/s)**	**ΔE**_**Q**_ **(mm/s)**
I) Fe_Ge_ - 6Te n.n.	Pure octa.	0.778	0.610	0.13
II) Fe_Ge_ - 4Te n.n.	Pure tetra.	0.020	0.090	0.00
III) Fe_Ge_ - 5Te n.n.	Defect-octa. (1 vacancy)	5.410	0.880	0.91
IV) Fe_Ge_ - 4Te n.n.	Defect-octa. (2 vacancies)	7.160	0.850	1.19
V) Fe_Te_ - 6Ge n.n.	Pure octa.	3.480	0.540	0.57
VI) Fe_Te_ - 4Ge n.n.	Pure tetra.	4.440	0.560	0.73

### Fe lattice site identification in GeTe

Following the implantation of radioactive ^57^Mn^+^ ions, the daughter Fe probe ions could in principle substitute for Ge (Fe_Ge_) and/or Te (Fe_Te_). Moreover, owing to the 〈*E*_*R*_〉 = 40 eV recoil energy imparted on the ^57*^Fe daughter nucleus in the *β* ^*−*^ decay of ^57^Mn, a fraction of the daughter ^57^Fe probe ions could be expelled from the initial site occupied by the implanted ^57^Mn ions to interstitial sites (Fe_I_)^45, 46^. Indeed, our eMS measurements on ^57^Mn/^57^Fe implanted Si and Ge^42, 47^ show appreciable interstitial fractions of the Fe ions. However, these studies also show that the Debye temperatures for substitutional Fe (Fe_Si_ and Fe_Ge_) extracted from the eMS resonance spectra agree well with estimates based on the mass defect approximation and those of interstitial Fe are at least 100 °C lower. In the present study, the average Debye temperature (*θ*_D_) for Fe in the GeTe samples, determined from the temperature dependence of the resonance area, is <*θ*_D_> = 175(25) K. This value is in good agreement with the value *θ*_D_ = 205 K for Fe substituting Ge estimated using the mass defect approximation, assuming a value *θ*_D_ = 180 K for the GeTe host lattice^48^. This allows us to exclude any significant contribution from interstitial Fe to the A, C and D components, as also confirmed by measurements on GeTe-1 (see Supplementary Information).

In principle, the ^57^Mn^+^ ions are expected to adopt the more electropositive site (Ge site). In the case of Fe substituting Te (Fe_Te_), the bonding with neighboring Ge atoms would require the charge state of Fe to lower to Fe^+^/Fe^0^, which would be expected to give a higher isomer shift than that measured for the A component (Table 1). Moreover, the preferential substitution of the cation as a dopant in group VI chalcogenides has been previously reported, with substitution of Te sites having a much higher formation energy^13, 49^. Liu *et al*. performed comparative XPS studies of Ge_1−x_Fe_x_Te films (x = 0.02–0.25) and FeTe^50^. The Fe 2p core-level XPS spectra revealed the two components Fe 2p_3/2_ and Fe 2p_1/2_, which were coincident in the GeFeTe and FeTe samples, indicating that Fe occupies substitutional Ge (cation) sites and is bonded with Te in the Fe incorporated GeTe films.

We now compare the measured *δ* and *ΔE*_*Q*_ for the A and C components (Table 1) with the respective values calculated for the different local configurations listed in Table 2. The hyperfine parameters of components C and A very well match those simulated for the I) and II) configurations in Table 2, respectively. We therefore assign the A component to the Fe_Ge_ - 4Te nn tetrahedral configuration in a-GeTe, and the C component to the octahedral Fe_Ge_ - 6Ge nn. The small calculated quadrupole splitting for the octahedral configuration I) in Table 2, is of the same order of magnitude as the additional line-width broadening observed for the C component in the eMS measurements (Table 1). The null quadrupole interaction in the tetrahedral configuration (A component) originates from the equal and opposite contributions to the electric field gradient given by the d(x^2^-y^2^) and dz^2^ orbitals^51^. The displaced Ge atoms take up interstitial sites probably diffusing upon thermal annealing. We exclude any incorporation of Ge in the immediate neighbourhood of Fe_Ge_ (nn or nnn), since this would readily generate a non-zero electric field gradient, and hence quadrupole splittings for the A and C components, which we do not observe.

The most debated question concerns the local structure of the a-GeTe phase^16–33^. In this context, a comparison of our results with Raman studies of a-GeTe is of interest. In particular, Andrikopoulos *et al*., have proposed a structure for a-GeTe that contains the tetrahedral configuration GeTe_4−n_Ge_n_, with n = 0, 1, 2, 3, 4^27^. Additional calculations of the Mössbauer parameters for the GeTe_4−n_Ge_n_ structure, with a combination of n = 0, 1, 2, 3 configurations in a 2 × 2 × 2 unit cell have been conducted (see Supplementary Information), yielding to *ΔE*_*Q*_ = 0.14 mm/s and *δ* = 0.17 mm/s. When compared to the Mössbauer parameters obtained for configuration II in Table 2 (i.e. n = 0), it is clear that our experimental data (A component, Table 1) are much better reproduced by the pure tetrahedral GeTe_4_ case.

It is evident from calculations (Table 2), that the introduction of one and two Te vacancies around Fe_Ge_ in the c-GeTe configuration strongly enhances the quadrupole splitting. In particular, configuration IV) in Table 2 with two Te vacancies yields *δ* and *ΔE*_*Q*_ values matching very well the experimental values for the D component (Table 1), which is characterized by a larger line broadening compared to components A and C (cf. Table 1). Consequently, we assign the D component to the defect-octahedral configuration proposed in ref. 33. In a-GeTe thin films, the fraction of defect-octahedral configuration has been shown to increase with the film thickness, and reported to be ≤30% for 100 nm layers^52^. By assuming the trend of the defect-octahedral fraction *vs* film thickness reported in ref. 52, we expect a fraction ≤35% in a 150 nm thick GeTe film. In GeTe-2, we detect a 60% of D fraction in a-GeTe (Fig. 4). Therefore, we conclude that our D component consists of two contributions: a ≤ 35% of Fe_Ge_ in the defect-octahedral configuration and a ≥25% fraction in a more disordered local configuration (distribution of bond angles and/or additional Te vacancies), due to the lattice damage induced by the implantation process. At the phase transition temperature of 180 °C, the ion-implantation induced damage is expected to disappear^42^. We therefore conclude that the ≤20% fraction of D component that is left in c-GeTe (Fig. 4) is due to a persisting fraction of Fe_Ge_ in the defect-octahedral configuration, with the remaining ≥20% fraction (A) due to tetrahedral, and the remaining 60% (C) due to octahedral sites. Results obtained by inelastic Raman light scattering on bulk c-GeTe^53^, report the local structure of crystalline GeTe as including a 16.7% of Ge atoms in tetrahedral configurations and a 29.9% in defective octahedra, in reasonable agreement with our findings.

By normalizing the fraction of Fe_Ge_ in a-GeTe only to the pure tetrahedral + defect-octahedral contributions (i.e. not considering the implantation-damage), we estimate fractions of ∼53% and 47%, respectively, for the two configurations. When compared with ref. 33 we could expect a lower amount of tetrahedral configuration. On the other hand, it is known that the tetrahedral fraction increases at lower thicknesses^52^. These values must be compared to the results of Raty *et al*.^54^, based on DFT simulations generated following the melt-quenched procedure. Raty *et al*. predict a fraction of 30% for the tetrahedral Ge atoms, lower than the 53% that we detect in a-GeTe. It is important to underline the importance of the difference between as-deposited and melt-quenched amorphous GeTe-based alloys in determining their atomic-scale structure. Indeed, it is typically reported a higher tetrahedral fraction in as deposited GeTe-based materials when compared to melt quenched counterparts^55–57^. This is due to the fact that the amorphization induced by laser or pulsed current (i.e. melt-quenched cases) forms a kind of intermediate structure between the as-deposited amorphous and crystalline phases, thus typically exhibiting a higher concentration of distorted octahedral Ge sites^55, 56^.

### Atomic-scale mechanisms of the amorphous-to-crystalline phase transition

Our results define a scenario in which the macroscopic structural (Fig. 2) and resistivity (Fig. 1) changes occurring in GeTe thin films at 180 °C, are connected to the local transformation at Fe_Ge_ sites *from:* a combination of pure tetrahedral (53%) and defect-octahedral (∼47%) configurations, *to:* a dominant pure octahedral structure (60%), with a residual fraction of ≥20% tetrahedral and ≤20% defect-octahedral sites.

Certainly, the weightiest effect across the phase change is the transformation of the pure tetrahedral to pure octahedral fraction (Fig. 3). This is in accordance with Raman studies, which have demonstrated that it is the n = 0 configuration in the amorphous GeTe_4−n_Ge_n_ that dominates the phase transition from a-GeTe to c-GeTe^27^.

Simultaneously to the structural change, there is an electronic charge transfer, which transforms the chemical bond character between Fe_Ge_ and neighbouring Te atoms. In particular, the measured isomer shift change between components A and C corresponds to an electronic charge transfer of approximately 1.6 *e/a*_0_ (*e* denotes the electronic charge and *a*_0_ = 0.53 Ǻ the Bohr radius) between Fe_Ge_ and neighbouring Te atoms, which takes place during the phase transition^34^. This electronic density variation at the Fe_Ge_ site is directly connected to a change in character of the chemical bonding, i.e. to the lowering of the covalence when transforming from a-GeTe to c-GeTe (Table 1 and Fig. 3). This is in accordance with the charge density calculations that confirm the higher degree of covalence along the Fe_Ge_-Te bonding in the case of local tetrahedral configuration of a-GeTe, when compared to the octahedral c-GeTe (Fig. 6). The change in covalence is due to the lower shielding originating from the *d*-orbitals in the tetrahedral a-GeTe configuration^34^, where a higher *p-d* hybridization is observed, when compared to the octahedral c-GeTe one. Kolobov *et al*. have shown that the energy-efficient phase transition in GeTe occurs through a bond switch, where the pairs of non-bonding valence *p*-electrons (residing in the same orbital and not participating in the formation of conventional covalent bonds) mediate the bond switch without the rupture of the strong covalent bonds of the amorphous state^21^. With eMS, we probe the chemical rearrangements occurring at the Ge site indirectly, i.e. through the hyperfine interactions experienced by Fe substituting Ge at the Fe_Ge_ site. It is not possible to compare quantitatively the electronic configuration changes across the Fe_Ge_-Te bonds with those of Ge-Te bonds due to the additional contribution of the *d*-orbitals to the chemical bond in the case of Fe_Ge_-Te. However, it is of much interest to attempt a comparison with ref. 21, since the experimental verification of the mechanism there proposed is still lacking and challenging. The small charge transfer of 1.6 *e/a*_0_ between Fe_Ge_ and the neighbouring Te atoms, as measured by eMS, is expected to be a particularly cost-effective process in terms of energy. Moreover, on the atomic-scale, a non-100% switch is evidenced from the local tetrahedral to the octahedral configuration: for the full thermal budget furnished to GeTe-2, which corresponds to a fully achieved macroscopic phase transformation (see Supplementary Information), there are still a ≥20% of the Fe_Ge_ atoms in the A-type spectral configuration (see Fig. 4). This is also the case of GeTe-1 (see Supplementary Information). The eMS results evidence that the macroscopic phase transition (Figs 1 and 2) is not accompanied by the full transformation of tetra – to – pure-octahedral configuration on the atomic-scale. We suggest that the coexistence of the A and C components (Fig. 3) following the phase transition, is a marker for the very delicate and *simultaneous* change of structure and chemical bonding around Fe_Ge_ during the macroscopic phase transition. It is therefore tempting to associate our experimental evidence with the energy efficient bond switch process proposed in ref. 21, and in particular with the suggested absence of a real rupture of the strong covalent bonds in a-GeTe following the phase transition.

There is an additional ≤20% of defect-octahedral Fe_Ge_ (component D) fraction that is left in c-GeTe, but this component does not show any change in its isomer shift, meaning it is not directly involved in the change of the chemical bonding. This demonstrates that the change in the nature of the chemical bond across the phase change is uniquely associated with the tetrahedral – to – pure-octahedral transformation.

### Summary

The macroscopic phase change and electrical conductivity switch occurring in GeTe at 180 °C were studied. A clear correlation with atomic-scale chemical-structural changes was established by monitoring the amorphous-to-crystalline phase transition by emission Mössbauer spectroscopy on ^57^Fe probes, substituting Ge in GeTe thin films.

Certainly, the most debated questions are: “*what is the local structure of a-GeTe and which mechanism drives the fast and reversible phase transition to and from c-GeTe*?” Our results show that the Ge environment in as-sputtered a-GeTe is a combination of tetrahedral (53%) and defect-octahedral (47%) configurations. With the experimental method applied here, employing the extreme sensitivity of the ^57^Fe probe, we followed *in situ* the local transformation occurring at Ge sites during thermal annealing. We show that the phase and resistivity changes characterizing the prototypical GeTe chalcogenide, are attributable to a local symmetry variation around Fe_Ge_ from tetrahedral and defect-octahedral (both surrounded by four Te atoms) in a-GeTe to octahedral (surrounded by six Te atoms) in c-GeTe (60%) with remaining fractions of ≥20% tetrahedral and ≤20% defect-octahedral sites, respectively.

Simultaneously, a small net-electron charge density transfer of ~1.6 *e/a*_0_ between the Fe_Ge_ and the neighbouring Te atoms was measured. This was found to be associated with the gradual change of the degree of chemical bonding from covalent to ionic. Most importantly, these chemical changes are uniquely associated with the transformation from the Fe_Ge_ tetrahedral fraction in a-GeTe to the local octahedral symmetry in c-GeTe, without any apparent involvement of the defect-octahedral fraction in a-GeTe. Our experimental results were corroborated by DFT calculations of the hyperfine parameters of the Fe probes in the different local symmetries.

## Methods

### Sample preparation

Amorphous 150 nm-thick Ge_50_Te_50_ stoichiometric thin films were deposited onto Si(550 μm)/SiO_2_(80 nm) substrates by DC magnetron sputtering of a GeTe target in Ar atmosphere. Two samples, labelled GeTe-1 and GeTe-2, cut from the same wafer, were the subject of the present study.

### GIXRD measurements

Grazing incidence X-ray diffraction (GIXRD) measurements were performed at an incidence angle ω = 1°, in order to investigate the crystalline structure of the crystals, prior to and following the thermal treatment and ion implantation. Measurements were performed with an upgraded XRD3000 (Italstructure) diffractometer with monochromated Cu Kα radiation (wavelength 0.154 nm) and a position sensitive detector (Inel CPS120).

### Resistivity measurements

The resistivity measurements on GeTe-1 were conducted during thermal annealing by using a four-probe setup in the Van der Pauw configuration. The sample was heated in contact with a heater-chuck, from RT to 250 °C and back to RT, at a constant rate of 10 °C/min, in a chamber which had been previously evacuated to <10^−5^ mbar, in order to prevent oxidation and contamination. The maximum temperature of 250 °C was chosen in order to ensure a complete GeTe crystallization.

### eMS measurements

eMS were conducted following the implantation of radioactive ^57^Mn^+^ (*T*_1/2_ = 1.5 min) ion beams at the ISOLDE facility at CERN. The beam was produced by 1.4 GeV proton-induced fission in UC_2_ targets and subsequent laser ionization^58^. Pure beams with intensities of ~5 × 10^8^ ions/s were implanted at 50 keV (fluence <10^12^ cm^−2^) into the GeTe sample held at temperatures from RT up to 210 °C in vacuum (10^−6^ mbar), in an implantation chamber. Under the implantation conditions reported here, the Mn ion range was estimated (TRIM) to be 32 nm. This rules out the possible effect of surface oxidation, which according to the X-Ray Reflectivity (not shown) is limited to 11 nm in GeTe-2. Each eMS spectrum was recorded following an average 5 min implantation and measurement time. Each sample received a maximum implantation fluence of ~1.5 × 10^12^ at./cm^2^, which is well below the threshold of overlapping damage cascades (typically 10^13^–10^14^ cm^−2^) in semiconductors and insulators^58^. Heating was performed with a halogen lamp mounted behind the sample. In the eMS experiments performed on GeTe-2, a temperature ramp rate of ~5 °C/min was used. ^57^Mn β-decays to the 14.4 keV Mössbauer state of ^57^Fe (*T*½ = 100 ns), allowing eMS spectra to be recorded using a resonance detector equipped with enriched ^57^Fe stainless steel electrodes, mounted on a conventional drive system outside the implantation chamber. The intrinsic line-shape and line-width of the detector were determined from implantations into an *α*-Fe foil, yielding a Voigt profile with Lorentzian line width (FWHM) of Γ = 0.34 mm/s and additional Gaussian-broadening of *σ* = 0.08 mm/s. Isomer shifts and velocities are given with respect to the centre of the spectrum of *α*-Fe at RT. The eMS spectra were analyzed by using the Vinda analysis program^41^.

### Calculation details

Theoretical calculations of the hyperfine interaction parameters were conducted by employing the generalized gradient approximation (GGA), within density functional theory (DFT). The full potential linearized augmented plane wave (FP-LAPW) method, as implemented in the WIEN2K code^59^, was employed together with the Perdew-Burke-Enzerhof (PBE) generalized GGA functional, for all of the DFT calculations^60^. In particular, simulations were done both with and without including the Hubbard-like Coulomb term U in the PBE parametrization. In the calculations, the considered radii of the muffin tin atomic spheres of Ge, Te and Fe were 2.3, 2.5 and 2.11 a.u., respectively. The atomic radii were chosen such that the mutual overlaps between all kinds of combinations of interstitial and atomic spheres are within the permissible limit of the atomic sphere approximation. Moreover, the distinction between the valence and core states was made through the energy value, and a value of -6 Ry was taken as the boundary separating the core electron states and valence electron states. The cut-off parameter in the calculations (R_MT_K_MAX_) was set to 7.0, a supercell size of 2 × 2 × 2 and a mesh of (4 × 4 × 4) k-points in the irreducible part of the first Brillouin zone were used in the GGA approximation. In this approach, the isomer shift *δ* and the quadrupole splitting *ΔE*_*Q*_ were calculated from their contact densities (*ρ*) and the principal component (*V*_*zz*_) of the electric field gradient, respectively, as reported in the literature^61^. In particular, the *ΔE*_*Q*_ is calculated in the axially symmetric electric field gradient approximation^34^.

The non-negligible hybridization between the *d*-valence band of Fe and the *p*-valence band of Te in the tetrahedral configuration, makes it necessary to include a Hubbard term U in the Coulomb interaction term in the GGA approximation. The U term is generally estimated by comparing calculated and measured physical properties. Assuming that U = 3 eV, the total magnetic moment of Fe_Ge_ in both the GGA and GGA + U approximations was calculated: for a-GeTe to be 0.90 and 2.35 respectively; for c-GeTe to be 2.38 without including the U term. The difference between a-GeTe and c-GeTe is due to the fact that in the local octahedral configuration (c-GeTe) the hybridization between *d* and *p* orbitals is lower than in the amorphous state. Therefore, even without inclusion of the U term, the total magnetic moment is close to the value for an isolated Fe atom.

### Data availability

The datasets generated during and/or analysed during the current study are available from the corresponding authors on reasonable request.

## Electronic supplementary material

Supplementary Information
