# Supplementary material for: Atomic-scale study of the amorphous-to-crystalline phase transition mechanism in GeTe thin films
Source: Sci Rep. 2017 Aug 15;7:8234. doi: 10.1038/s41598-017-08275-5 (PMC5558007; doi:10.1038/s41598-017-08275-5)
Supplement: Supplementary file 1 — Supplementary Information [file 41598_2017_8275_MOESM1_ESM.doc]

SUPPLEMENTARY INFORMATION to the paper:

Atomic-scale study of the amorphous-to-crystalline phase transition mechanism in GeTe thin films

by R. Mantovan, R. Fallica, A. Mokhles Gerami, T. E. Mølholt, C. Wiemer, M. Longo,

H. P. Gunnlaugsson, K. Johnston, H. Masenda, D. Naidoo, M. Ncube, K. Bharuth-Ram,

M. Fanciulli, H. P. Gislason, G. Langouche, S. Ólafsson and G. Weyer

**Thermal budget to achieve full crystallization in GeTe**

In GeTe-2, following the phase transition to the c-GeTe state, still about a 20% of Fe atoms are found in the A configuration (after the measurement (d) in Fig.3, main text), therefore sensing the local environment typical of the (macroscopic) a-GeTe. On the other hand, the A fraction that is left in the crystalline GeTe-1 (Fig.3, main text) is 5.5%. This suggests a more efficient amorphous-to-crystalline transition in GeTe-1 than in GeTe-2, most probably due to the different annealing set-ups that are used to induce the phase transition in GeTe-1 (Fig.1, main text) and GeTe-2 (Fig.3, main text), see Section II. In GeTe-1, the crystallization state is accurately detectable as a change in electrical resistivity of the former sample during its heating in direct contact with a hot plate; whereas this is not trivial in GeTe-2, which is annealed in the eMS chamber by means of a halogen lamp (see Section II, main text). Indeed, since the amorphous-to-crystalline transition of a chalcogenide is a thermodynamical process of the second order, its rate depends non-linearly on the thermal history *T(t)* it experiences. It is assumed that the crystallization rate is the product of two terms: the first depending on temperature T, while the second is a function of the crystallized portion of material. The T-dependent term follows an Arrhenius-type exponential law, with activation energy of 3 eV (previously determined by constant heating rate experiments, not shown). From the thermal history *T(t)* of sample GeTe-2, we estimated the thermal budget (TB) received by the material at the different stages of annealing during the eMs measurements reported in Fig.3(a)-(d) of the main text. The thermal history and corresponding thermal budget are shown in Fig. S1, where the dots with the letters indicate the corresponding eMS spectra depicted in Fig.3(a)-(d) of the main text.

Fig. S1. Thermal history *T(t)* of sample GeTe-2 during the on line eMS experiments at ISOLDE/CERN. Green line: the thermal budget received by the material at the subsequent eMS measurements reported in Fig.3(a)-(d) of the main text.

It is evident that, at the first two implantations/measurements, the TB given to the sample is practically zero, Figs. 3(a) and (b) in the main text. At the third implantation/measurement at 210°C, Fig. 3(c) in the main text, the TB given to the sample rapidly increases, remaining constant at t ≥ 50 min, where the full thermal budget is finally furnished to the sample.

**eMS in GeTe-1**

Figure S2 shows eMS in GeTe-1 at 37 °C and 150 °C. The spectra well resemble those reported in Fig. 3(c,d) of the main text, as expected for c-GeTe. The results also confirm that the A🡪C transition lines cannot originate from interstitial Fe becoming substitutional above 180 °C. Already in the as implanted state (Fig. S2(b)), the shape of the spectrum shows the dominant C single line component and a left fraction (7.5 %) of the A component, which cannot therefore be interpreted as originating from recoil-produced interstitial Fe in GeTe. Annealing of GeTe-1 at 150 °C (Fig. S2(a)) favours the disappearance of component A together with the annealing of the component D, with a left 24% fraction.


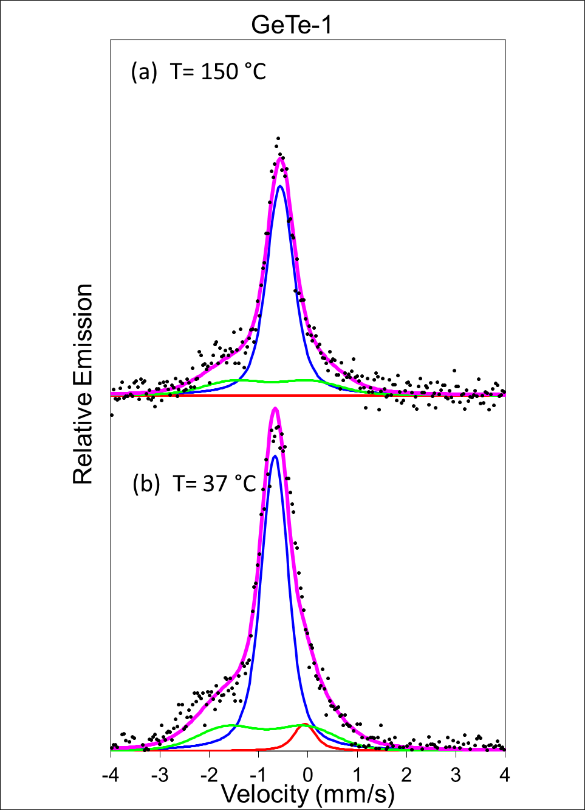


Fig. S2. eMS spectra at (a) 150 °C and (b) 37 °C in GeTe-1.

The reason for the absence of interstitial fraction in both GeTe-2 (main text) and GeTe-1, could be either a much higher threshold energy for displacement of the Fe ions from their initial implantation site when compared to the Mn🡪Fe recoil energy, or the incorporation of interstitial Fe on regular lattice sites within the lifetime of the 14.4 keV ^57^Fe Mössbauer state (140 ns).

**DFT of the Fe_Ge_ in c-GeTe with 1 and 2 Te vacancies**

In order to simulate the presence of 1 and 2 Te vacancies around Fe_Ge_, we started from the configuration of Fe_Ge_ surrounded by 6 Te in the c-GeTe structure, and removed 1 and 2 neighboring Te as depicted in Fig.S3.

(a)
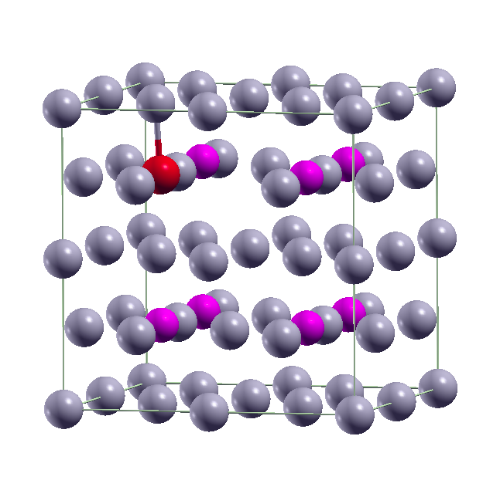


(b)
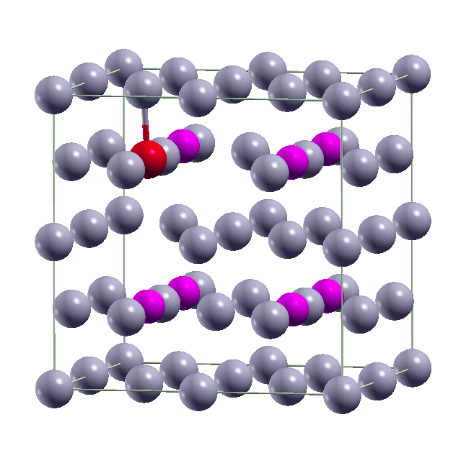


Fig. S3. Local structure around Fe_Ge_ with (a) additional one and (b) two Te vacancies in a 2x2x2 supercell of c-GeTe

The periodic boundary conditions are the same for all configurations (those in Fig.S3 and in Fig. 5 in the main text): the lattice constant is 6.02 Å, and the mesh of 4x4x4 k-points in the irreducible part of the first Brillouin zone has been used in the GGA approximation. The symmetry of unit cell is different for each configuration due to the presence of vacancies.

**DFT for a distribution of GeTe_4-n_Ge_n_ with n=0,1,2,3**

In order to compare our results with those obtained by Raman in a-GeTe [Andrikopoulos, K. S. et al., Raman scattering study of the a-GeTe structure and possible mechanism for the amorphous to crystal transition, *J. Phys. Condens. Matter.* **18**, 965 (2006).], we conducted additional calculations of the Mössbauer parameters for the GeTe_4-n_Ge_n_ structure, with a combination of n=0,1,2,3 configurations, as depicted in Fig.S4.


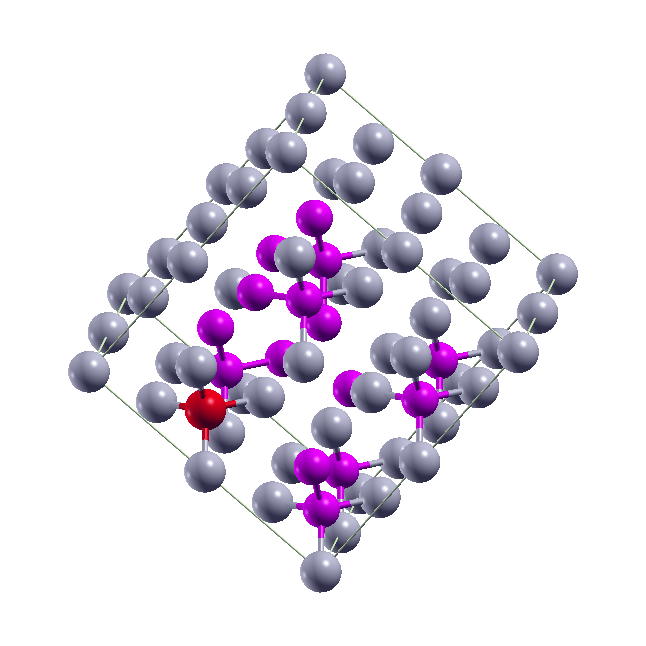


Fig. S4. Combination of a variety of GeTe_4-n_Ge_n_ tetrahedral units, with n=0,1,2,3 in a 2x2x2 supercell. Red, purple and gray spheres indicate Fe, Ge and Te atoms respectively.

The obtained averaged hyperfine parameters calculation yield *ΔE_Q_* = 0.14 mm/s and *δ* = 0.17 mm/s.
